# The influence of high-level beliefs on self-regulatory engagement: evidence from thermal pain stimulation

**DOI:** 10.3389/fpsyg.2013.00614

**Published:** 2013-09-23

**Authors:** Margaret T. Lynn, Pieter Van Dessel, Marcel Brass

**Affiliations:** ^1^Department of Experimental Psychology, Ghent UniversityGent, Belgium; ^2^Department of Experimental Clinical and Health Psychology, Ghent UniversityGent, Belgium; ^3^Behavioral Science Institute, Radboud UniversityNijmegen, Netherlands

**Keywords:** free will, beliefs, inhibition, volition, effort, self-control, pain

## Abstract

Determinist beliefs have been shown to impact basic motor preparation, prosocial behavior, performance monitoring, and voluntary inhibition, presumably by diminishing the recruitment of cognitive resources for self-regulation. We sought to support and extend previous findings by applying a belief manipulation to a novel inhibition paradigm that requires participants to either execute or suppress a prepotent withdrawal reaction from a strong aversive stimulus (thermal pain). Action and inhibition responses could be determined by either external signals or voluntary choices. Our results suggest that the reduction of free will beliefs corresponds with a reduction in effort investment that influences voluntary action selection and inhibition, most directly indicated by increased time required to initiate a withdrawal response internally (but not externally). It is likely that disbelief in free will encourages participants to be more passive, to exhibit a reduction in intentional engagement, and to be disinclined to adapt their behavior to contextual needs.

## Introduction

The question of whether free will truly exists is an age-old philosophical question, tackled by thinkers ranging from Democritus to Russell. Yet most contemporary scientists have avoided the metaphysical and existential hurdles of free will, and instead investigate its impact on human action: how this phenomenon arises in the mind, and to what extent deterministic beliefs have an effect on our behavior (e.g., Wegner, 2003; Vohs and Schooler, 2008; Baumeister et al., 2009; Rigoni et al., 2011, 2012, 2013). The sensation of control over one's actions is an undeniably ubiquitous feature of human experience. People tend to believe they are responsible for a given action if the causal principles of *consistency, priority*, and *exclusivity* are satisfied that is, if their intentions are consistent with and experienced at a suitable interval prior to the relevant action, and there is no other reasonable explanation for the action arising (Wegner, 2003). Perception of personal control is further considered to be intrinsic, biologically necessary, and protective against environmental stressors (Leotti et al., 2010).

Social psychological research has recently investigated the degradation of behavioral and social effects thought to follow from a belief in determinism. For instance, Vohs and Schooler (2008) found that inducing disbelief in free will, via reading of a determinist essay or series of statements, elicited an increase in cheating on the part of participants. In comparison with control subjects, anti-free will participants in this case paid themselves a statistically improbable amount of money for performance on a problem-solving task, and more frequently permitted themselves to view answers when given the opportunity to cheat. Under similar conditions, Baumeister et al. (2009) found that participants with weakened free will beliefs showed increased aggression and decreased helping behavior. Likewise, an increase in mindless conformity and a decrease in counterfactual thinking, assumed to be adaptive for learning and social adaptation, have been reported to accompany deterministic beliefs (Baumeister et al., 2011; Alquist et al., 2013). Interestingly, when these studies included a condition promoting free will, results were consistent with the control group, suggesting that a belief in free will is a common default state.

More recent research in the domain of Cognitive Psychology has revealed an impact of deterministic beliefs even on basic levels of motor control. Rigoni et al. (2011) used a manipulation identical to that of Vohs et al. (2008, Experiment 1) to alter participants' belief in free will. They observed that participants who were induced to disbelief in free will showed reduced amplitudes of the readiness potential, an electrophysiological marker of unconscious motor preparation (Rigoni et al., 2011). In a subsequent study (Rigoni et al., 2013) it was found that performance monitoring, as indicated by post-error slowing, was also diminished in participants induced to disbelieve in free will. This may indicate a reduction in the recruitment of self-regulatory processes, and less inclination to adjust one's behavior according to circumstantial needs, on the part of anti-free will participants.

Finally, this belief manipulation has been applied to an important facet of self-control, namely *intentional inhibition*, or the ability to voluntarily suppress a prepotent action plan (Brass and Haggard, 2007). The study in question (Rigoni et al., 2012) employed a task developed by Kühn et al. (2009) that overcame a limitation of the well-supported literature on externally-generated stopping (see Aron, 2007, for a review) by enabling voluntary choice behavior to be experimentally investigated within an inhibition paradigm. In this task, participants were occasionally asked to freely decide whether to stop a prepared action (button pressing to halt the progress of a marble rolling down a ramp). Both intentional inhibition and perceived self-control were shown to be adversely affected by an anti-free will manipulation (Rigoni et al., 2012). These findings were interpreted such that weakened free will beliefs lead to a reduction in intentional effort, which then causes participants to select the less demanding response option (in this case to execute the pre-planned response).

The goal of the present study was to support and extend prior research on the influence of free will beliefs upon intentional inhibition, by investigating whether inducing determinist beliefs might in turn influence one's intentional engagement in self-regulatory behavior. However, while previous studies have investigated intentional inhibition in rather artificial experimental situations in which participants have hardly any prior motivation to act or inhibit, we sought to address voluntary inhibition in a more ecologically valid setting in which behavioral urges are present. To this end, our secondary goal was to develop and pilot a novel experimental paradigm for disentangling intentional from instructed inhibition.

Pain was selected as the behaviorally relevant stimulus for our purposes. Management of the pain avoidance response can be seen as a compelling component of the affective response system; the organism is strongly motivated to avoid the pain sensation (Campbell and Misanin, 1969; Elliot, 2006). We can therefore consider management of this urge as a window into how we suppress our most basic drives, and a classical instance of self-control. The pain avoidance response can of course be highly automatized, for instance when one reflexively jerks their hand away from a hot stove. However, at times other goals call for self-control to be exerted for the suppression of this avoidant urge, such as when the heat comes not from the stove, but from a plate of food. In this case, one might choose to suppress the highly prepotent reaction momentarily in favor of satisfying the opposing basic urge of hunger (cf. Morsella, 2005).

Our paradigm required participants to occasionally inhibit a prepotent withdrawal reaction from a heat source applied to their inner wrists. In half the trials, participants were able to choose whether to inhibit the withdrawal response or to immediately terminate the trial. The advantage of this manipulation is that it requires strong (and consistent; the urge to withdraw does not fade) self-control to withstand the thermal pain. In that sense, it is in stark contrast to standard laboratory tasks involving self-regulation and agency. The design also ensures that acting and inhibiting were equally distributed in the non-choice, or directed, trials, thereby discouraging any response bias and ensuring a comparable number of trial in each design cell. To manipulate free will beliefs, we used a Velten procedure (Velten, 1968) similar to that used in previous experiments (Vohs and Schooler, 2008, Experiment 2; Baumeister et al., 2009), in which participants are required to read and reflect upon a series of statements (see Supplementary Material for a complete list). Immediately prior to each trial, participants were presented with a statement and asked to retain the statement in memory until the end of the block. Statements were either neutral or meant to induce anti-free will beliefs (between-subjects). These statements were shown during the inter-trial interval in order to reduce potential pain preparation and decision-making strategies. We hypothesized that inducing disbelief in free will would lead participants to exhibit a reduction in intentional engagement, to lack adaptive strategies, and to be disinclined to adapt their behavior to contextual needs.

## Methods

### Participants

Fifty-four Dutch-speaking undergraduate students enrolled in the study; all gave written consent prior to participation. They received either course credit or a payment of 10 euros for their participation. All participants had normal or corrected-to-normal vision and reported no neurological deficits. The study was conducted in accordance with the Declaration of Helsinki, and the approval of Ghent University's Ethical Committee was obtained in advance. After determining participants' individual pain thresholds, those who did not report sufficient pain (i.e., their threshold surpassed 50°—beyond the safety limitations of the stimulating equipment) were removed from the study. A total of 48 participants (12 male, tested individually) completed the entire experiment.

### Procedure

#### Threshold determination

Pain was induced via a thermode connected to a Medoc PATHWAY device (MEDOC, Haifa, Israel), an apparatus designed to induce thermal pain using cold or hot stimulation. The threshold at which participants felt sufficient pain was determined by exposing each participant to 26 trials in which the thermal sensation gradually increased over 5 s from 32°C to a randomized destination temperature between 45 and 50°C (in increments of 0.25°), a slope comparable to the experimental trials. After each trial, the thermode returned instantly to baseline temperature, and participants were asked to rate their perceived pain on a scale from zero to eight, with zero being no pain and eight being the worst possible pain. The destination temperature employed in the main experiment was computed for each participant as the highest temperature at which they rated their pain as a six. This method was revealed during piloting to yield more accurate tolerance threshold measurements than merely requiring participants to indicate the maximum heat they could withstand when exposed to a steadily increasing temperature. Importantly, participants were free to press a button at any point during the threshold determination in order to terminate the trial.

#### Task and stimuli

Participants received painful heat stimulation during each trial, applied via a thermode to alternating inner wrists. The images of three geometric shapes (triangle, square, circle) were used as cues to indicate the trial type. Depending on the cue, participants were requested to either press the button as quickly as possible (“directed action,” 25% of trials), inhibit this response and endure the pain (“directed inhibition,” 25% of trials), or make a voluntary decision to either button press immediately or persist until the end of the trial (“choice,” 50% of trials). In the latter case, participants were requested to make their choices approximately equal over the course of the experiment, but not to use any particular strategies or to decide in advance of the presentation of the cue. In a practice block, absent pain stimulation, participants were trained on the cues. A pilot study had revealed that participants are typically around 200 ms slower to respond on choice action trials than on directed action trials, reflecting the additional time needed for the choice decision. Accordingly, to make stimulation as identical as possible across action conditions, 200 ms of thermal stimulation was added to directed action trials, following the button press.

**Figure 1:**
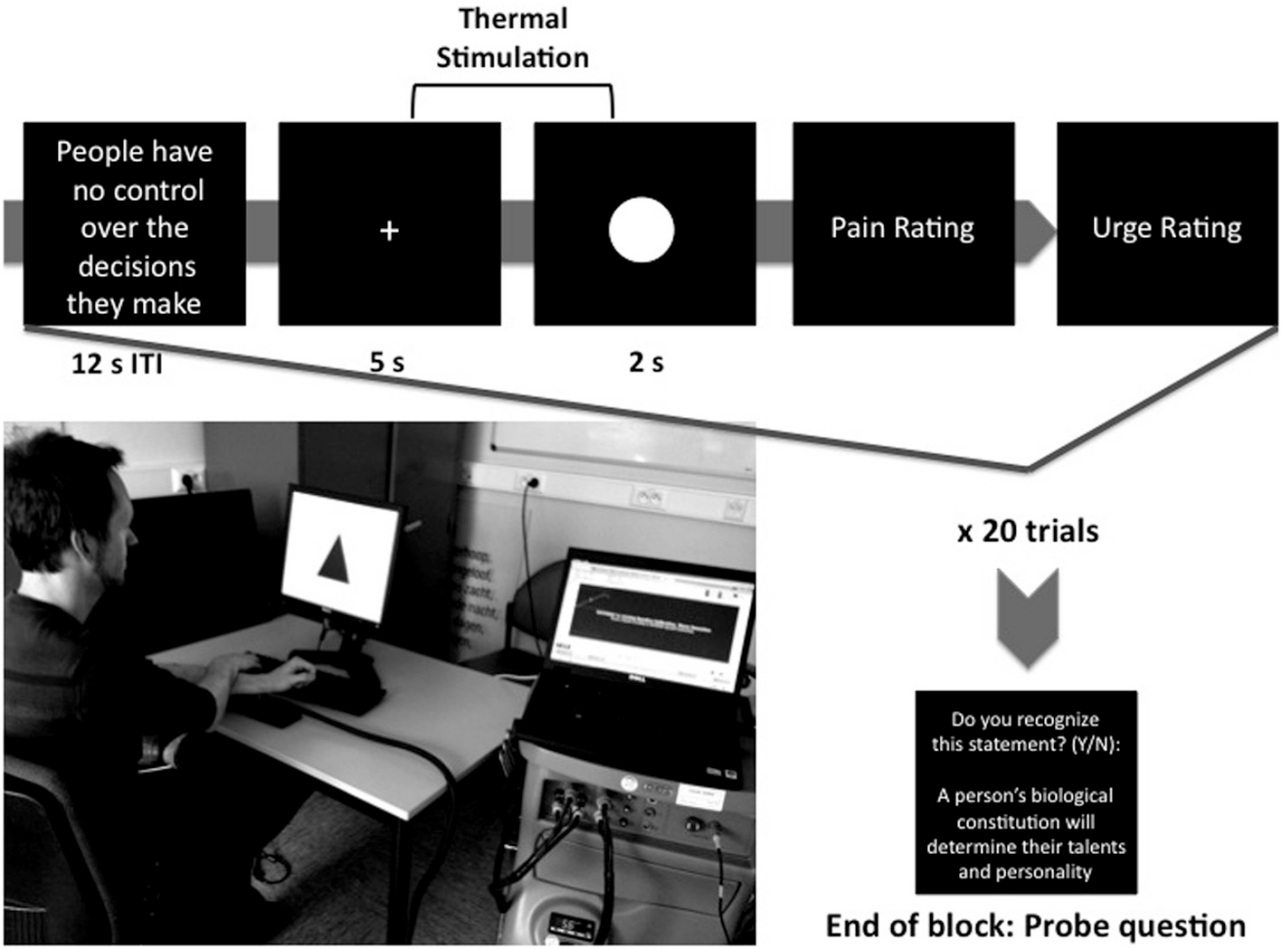
**Schematic overview of a sample block (Anti-free will condition)**. Note that there was no time limit for pain and urge rating responses.

Each trial was preceded by a statement (“neutral” or “anti-free will,” see below) with a duration of 12 s. After a delay of 1 s, a fixation cross was presented and the temperature of the thermode began to gradually increase from a baseline of 32°C to the participant's individually determined threshold. After 5 s, one of the three task cues appeared in place of the fixation cross. The temperature remained at threshold for the next 2 s, or until the participant pressed the button to terminate both the pain stimulation and the trial. Afterwards, prompts for ratings of the perceived pain and “urge to terminate the trial by pressing the button” (both on a scale of 0–8) remained on screen until participants responded. Participants were then cued to alternate the arm placed atop the thermode. The arm not being stimulated was used to button press (thereby providing a response time for action trials) and was placed atop the opposing wrist, in order to lend weight and make it more difficult for participants to inadvertently withdraw from pain rather than button pressing. A schematic overview of a possible trial in the anti-free will condition is presented in Figure 1.

The assignment of geometric shapes to trial types, and the order of the first-stimulated wrist were counterbalanced across subjects. Each participant had to perform 120 trials in total, being divided into six blocks of 20 trials presented in randomized sequence. In each block, participants were given 10 trials in which they were cued to make a decision, five trials in which they were cued to push and five trials in which they were cued to inhibit their withdrawal response. Importantly, participants were free to press a button to immediately terminate the thermal sensation at any point during the experiment.

#### Manipulation of free will beliefs

Participants were randomly assigned to either the control condition or the anti-free will condition (24 in each condition). All participants were required to read discrete statements presented on-screen during the inter-trial interval. They were instructed to retain this information until the end of the block, at which point a probe question concerning statement recognition was presented on the screen (see Supplementary Material). The probe questions were inserted to verify that participants had attended to the statements as directed, and to support a cover story that the study's goal was to test the influence of pain on memory. After feedback on the accuracy of their answer was given, a novel set of statements was presented, and subjects were instructed to remember these subsequent statements instead. The statements were either neutral or designed to tap into free will beliefs, with 60 unique statements in each group. Over the course of the experiment, control participants were exposed to each of the 60 neutral statements twice, while participants in the anti-free will condition were shown each of the 60 statements related to free will beliefs twice. Furthermore, in the anti-free will condition, the three trial types (directed action, directed inhibition, choice) were divided equally over each of the three statement categories.

A total of 90 statements were collected from a variety of questionnaires and articles involving free will beliefs (e.g., Carey, 2005; Vohs and Schooler, 2008; Paulhus and Carey, 2011), or were produced based on these inventories. These 90 statements were selected with the aim of being related to certain aspects of free will beliefs; 30 statements were related to the idea that people do not have a free will (e.g., “scientists tell us that people have no free will”), thirty statements concerned beliefs in scientific determinism (e.g., “the environment someone is raised in determines their success as an adult”) and 30 statements were related to beliefs in fatalistic determinism (e.g., “you can't change your destiny, no matter how hard you try”). Another 90 neutral statements were selected, stating facts and ideas that were unrelated to beliefs in free will (e.g., “an ostrich's eye is bigger than its brain”).

The combined 180 statements were then rated online (http://www.thesistools.com) by 38 participants, none of whom participated in the main experiment. Participants rated how difficult they would find the statement to recall, and the degree to which the statement was in line with either a disbelief in free will, a belief in scientific determinism, or a belief in fatalistic determinism. These questions were based on the factors laid out by Paulhus and Carey (2011) and were expressed in layman's terms for ease of understanding.

A total of 120 statements were selected based on the ratings drawn from this pre-test. The 20 statements that had received the highest ratings in each belief category were chosen, for a total of 60 experimental statements. Sixty neutral statements were matched for difficulty with these statements. Crucially, the experimental statements and the control statements did not differ with regard to their difficulty to recall (experimental: *M* = 1.59; neutral: *M* = 1.60), *t*_(7)_ = 0.86, *p* = 0.82.

#### Questionnaires

Two days prior to their participation in this study, participants completed an array of questionnaires concerning memory, anxiety, and free will beliefs. Questions about memory and anxiety were inserted to support the aforementioned cover story. Questions regarding free will beliefs consisted of the entire battery of the Free Will and Determinism questionnaire (FAD-Plus, Paulhus and Carey, 2011). Following the experimental session, participants were requested to complete the FAD-Plus questionnaire a second time to determine whether or not the experimental statements had an effect on the relevant belief system.

## Results

### Manipulation check

**Figure 2:**
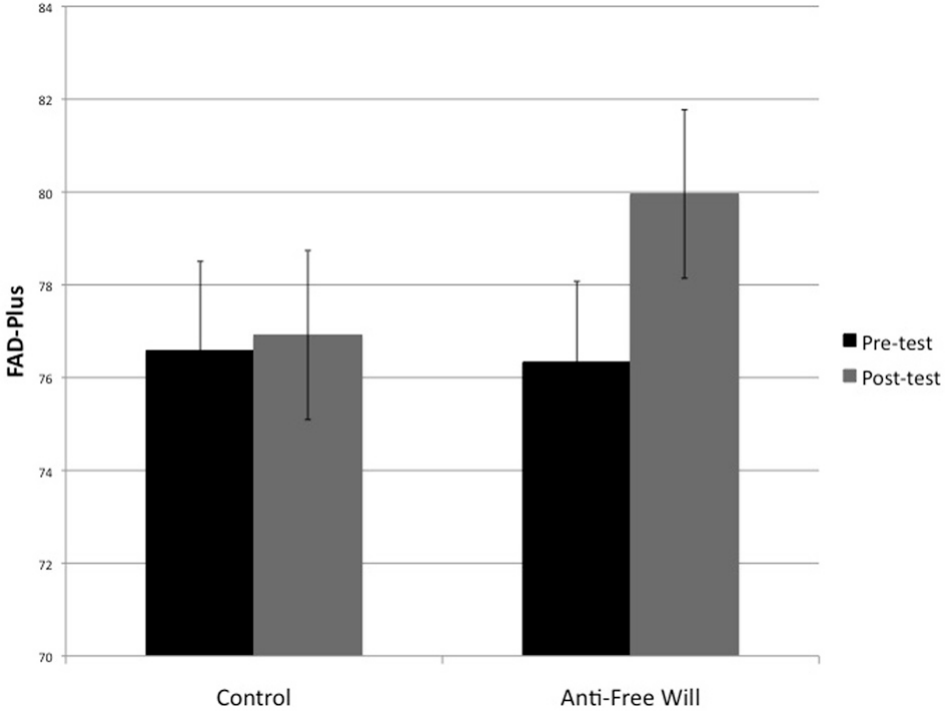
**Mean total scores on the FAD-Plus questionnaire as a function of Belief condition (Control vs. Anti-free will) and Time (Pre-test vs. Post-test)**. Higher scores indicate increased disbelief in free will.

To test the effectiveness of the belief manipulation, a mixed design ANOVA was conducted on participants' total FAD-scores before and after the experiment using Time (Pre-test vs. Post-test) as a within-subject factor and Belief condition (Anti-free will vs. Control) as a between-subjects factor. Total FAD-scores were calculated for each participant such that higher values indicate less belief in free will, by reverse scoring the Free Will subscale and combining it with the other three subscales (Scientific Determinism, Fatalistic Determinism, and Unpredictability). The analysis revealed a significant interaction between Time and Belief Condition, *F*_(1, 46)_ = 4.19; *p* < 0.05 (Figure 2), such that participants in the experimental condition scored significantly higher after the experiment than before (Post-test: *M* = 80.0, *SD* = 8.9; Pre-test: *M* = 76.3, *SD* = 8.5), *t*_(23)_ = 3.23, *p* < 0.01, indicating a weakening of beliefs in free will. No such effect was observed for participants in the control condition (Post-test: *M* = 76.9, *SD* = 8.9; Pre-test: *M* = 76.6, *SD* = 9.4), *t*_(23)_ = 0.29, *p* = 0.78.

### Data preparation

Despite efforts toward optimizing the pain threshold procedure, the grand mean pain rating across participants was rather low (*M* = 4.6; *SD* = 1.11). Crucially, in the debriefing questionnaire, more than half (*N* = 26) of all participants stated that they had not needed to exert any effort to withhold the pain-withdrawal response during the experiment. As pain is a key factor in this experiment, we decided to restrict our analyses to participants that reported a sufficient level of pain throughout the whole of the experiment. We therefore excluded all participants with mean pain ratings lower than the median of the subjective pain scale, namely 4.5. All further analyses were performed on this subset of 25 “high pain” participants (8 male): 12 participants in the anti-free will condition and 13 participants in the control condition. Results for the excluded “low pain” participants may be found in Supplementary Material.

### Behavioral analyses

Between-group means and standard deviations are reported in Table 1.

**Table 1:** **Between-group means and standard deviations**.

	**Control**		**Anti-free will**	
	**Mean**	***SD***	**Mean**	***SD***
**Reaction times (ms)**				
All action trials	658	124	736	101
Choice action trials	748	162	871	133
Directed action trials	552	121	582	94
Proportion inhibition (%)	40.59	9.64	42.43	10.22
Pain ratings (across trials)	5.5	0.9	5.4	0.6
**URGE ratings**				
Across trials	4.4	1.3	4.7	1.5
Choice trials	4.5	0.4	4.5	0.4
Directed trials	4.3	0.4	4.8	0.3

#### Reaction times

**Figure 3:**
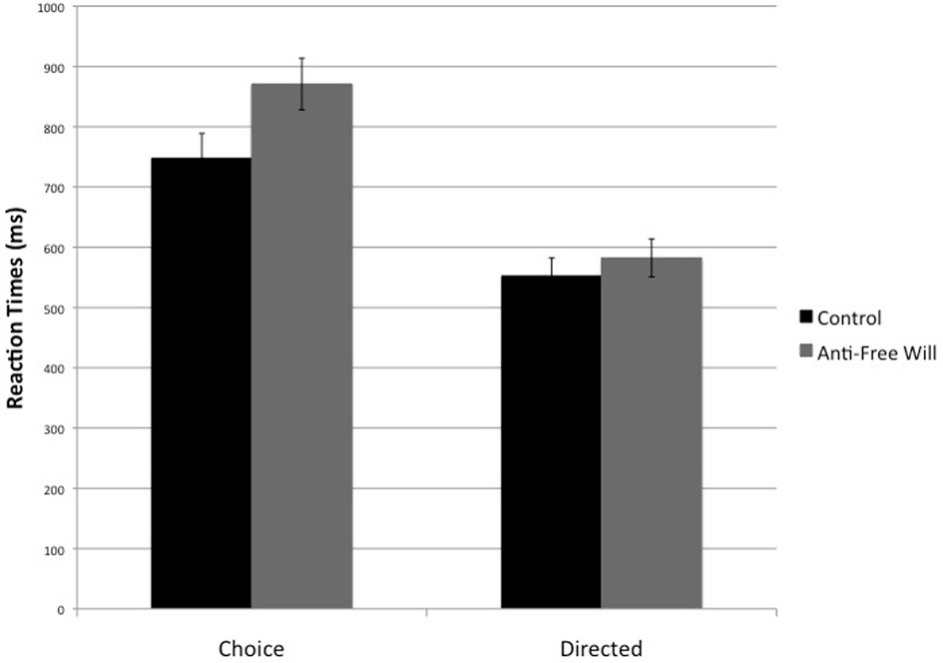
**Reaction times on press trials, between-subjects**. Note: values depicted are means and standard errors.

On trials in which participants were cued to button press, participants performed the correct response in nearly all trials (*M* = 99%, *SD* = 2%). We expected anti-free will participants to be significantly slower than controls, particularly on choice trials. A mixed design ANOVA on RTs, with Instruction (Choice vs. Directed) as a within-subjects factor and Belief condition (Anti-free will vs. Control) as a between-subjects factor, revealed a main effect of Instruction, *F*_(1, 23)_ = 79.310, *p* < 0.01, such that participants were slower to respond on choice trials (Choice: *M* = 807 ms, *SD* = 158 ms; Directed: *M* = 567 ms, *SD* = 108 ms), consistent with piloting and reflecting the time needed for a response decision. A main effect of Belief condition revealed a non-significant trend, *F*_(1, 23)_ = 2.958, *p* = 0.099, indicating that anti-free will participants tended to be slower to respond than controls (though this interpretation should be approached with caution due to the marginal significance level). Further, the interaction between Instruction and Belief condition trended toward significance, *F*_(1, 23)_ = 2.928, *p* = 0.10. Planned comparisons revealed an RT difference between anti-free will participants and controls on choice action trials, *t*_(23)_ = −2.07, *p* < 0.05, Cohen's *d* = 0.84 (Figure 3), such that anti-free will participants were significantly slower to respond when given a choice than were controls. No such effect was found on directed action trials, *t*_(23)_ = −0.69, *p* = 0.497, *d* = 0.27.

#### Correlation of FAD difference scores with choice reaction times

**Figure 4:**
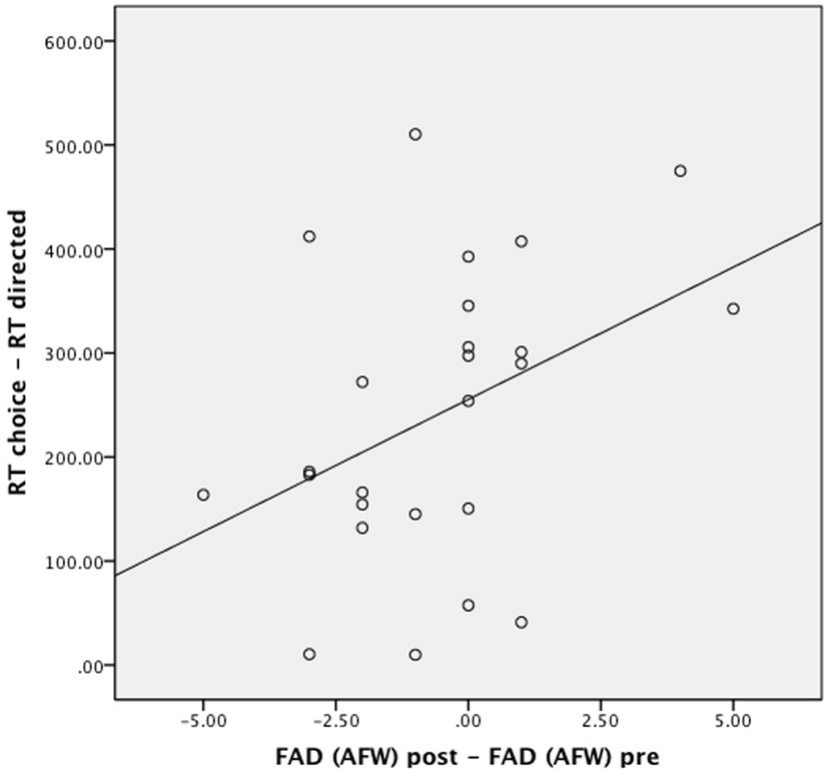
**Correlation of difference scores (post-test minus pre-test) on the anti-free will subscale of the FAD-Plus with the decision response time index (mean response times on choice minus directed trials)**.

To examine the relationship between participants' RTs and free will beliefs more thoroughly, we performed an additional correlation analysis. The aim of this analysis was to test to what extent the slowed responding on choice action trials was related to the effectiveness of the belief manipulation. To this end, we first computed each participant's change in anti-free will beliefs, across experimental condition (control participants were included to ensure sufficient variability), by subtracting participants' post-experimental scores on the anti-free will subscale of the FAD from their pre-experimental scores. Second, we computed a difference score of participants' mean RTs on choice and directed action trials to create an index of each participant's decision time at pushing the button. There was a significant positive correlation between the two difference scores, *r*_(23)_ = 0.40, *p* < 0.05 (Figure 4), reflecting that those subjects who showed a stronger reduction in free will beliefs were also slower to make the decision to press the button.

#### Proportion of inhibition on choice trials

On trials in which participants were cued to choose between acting and inhibiting, participants opted to inhibit in 41.47% of all trials (*SD* = 9.76%). The proportion of inhibition on choice trials was analyzed in an independent-samples *t*-test, revealing no significant difference between anti-free will participants and controls, *t*_(23)_ = −0.462, *p* = 0.648. This lack of a difference between experimental groups, which is in contrast to the findings of Rigoni et al. (2012), may be due to the experimental design, which, unlike previous studies, discourages response biases by using an equal proportion of directed action and inhibition trials.

### Ratings

#### Pain ratings

We began by computing pain ratings across all participants for the first and second halves of the experiment to ensure that participants did not adapt to the pain stimulation over the course of the experiment. No differences in pain ratings were observed between the trials of the first and the second half of the experiment (First half: *M* = 5.4, *SD* = 0.8; Second half: *M* = 5.5, *SD* = 0.8), *t*_(24)_ = −0.58, *p* = 0.57.

Participants reported a grand mean pain rating of 5.5 (*SD* = 0.74). Pain ratings were analyzed in a mixed design ANOVA using Belief condition as a between-subjects factor, and Response (Action vs. Inhibition) and Instruction (Directed vs. Choice) as within-subject factors. The main effect of Belief condition was not significant, *F*_(1, 23)_ = 0.13, *p* = 0.73, reflecting that subjective pain across trials was equivalent for the two groups. However, there was a significant main effect of Response (Action: *M* = 5.3, *SD* = 0.2; Inhibition: *M* = 5.7, *SD* = 0.1), *F*_(1, 23)_ = 12.60, *p* < 0.01, indicating higher perceived pain on inhibition compared with action trials, presumably due to the lengthier pain stimulation. Moreover, there was an interaction effect of Response × Instruction, *F*_(1, 23)_ = 7.94, *p* = 0.01, reflecting that inhibition trials were rated as less painful when they were voluntarily chosen rather than instructed (Choice: *M* = 5.5, *SD* = 0.8; Directed: *M* = 5.8, *SD* = 0.6), *t*_(24)_ = 3.38, *p* < 0.01, while there was no such difference between chosen and directed action trials (Choice: *M* = 5.4, *SD* = 1.0; Directed: *M* = 5.2, *SD* = 0.9), *t*_(24)_ = −1.54, *p* = 0.14. Importantly, the lack of a difference between the mean pain ratings of anti-free will and control participants suggests that our findings are not solely due to differences in the overall subjective experience of pain.

#### Urge ratings

**Figure 5:**
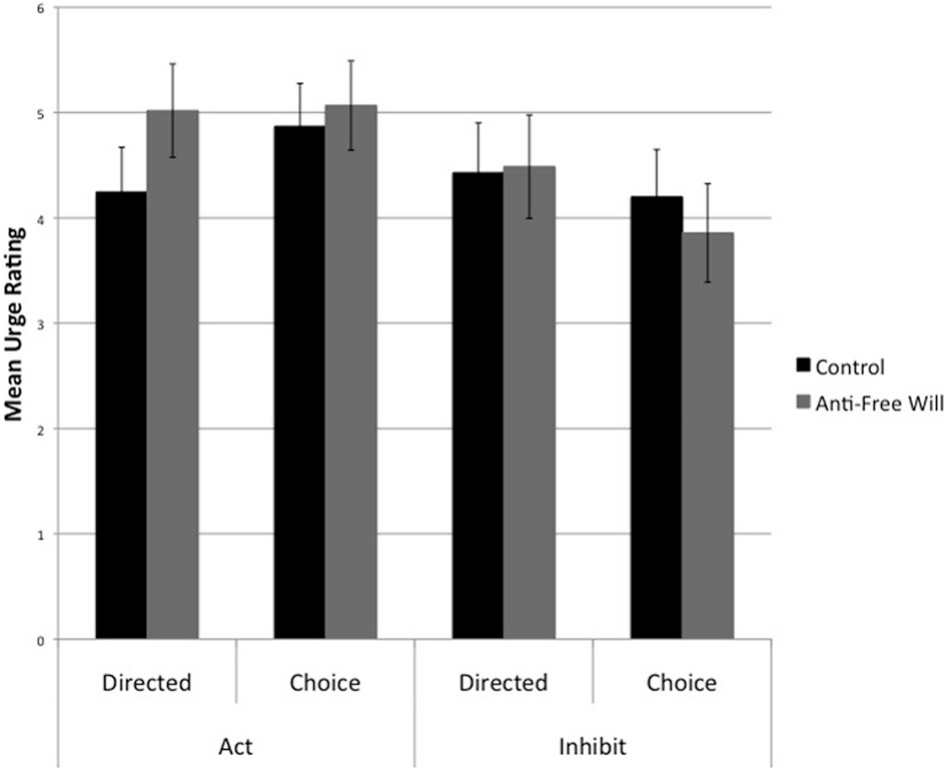
**Urge ratings as a function of Instruction (Choice vs. Directed) and Belief condition (Control vs. Anti-free will)**. Note: values depicted are means and standard errors.

Participants reported a grand mean urge rating of 4.5 (*SD* = 1.4). Urge ratings were analyzed with a mixed design ANOVA akin to that of the pain ratings. The analysis revealed a significant main effect of response, reflecting greater urges on action trials (Action: *M* = 4.8, *SD* = 0.3; Inhibition: *M* = 4.2, *SD* = 0.3), *F*_(1, 23)_ = 4.98, *p* < 0.05. There was also a significant interaction effect of Response × Instruction, *F*_(1, 23)_ = 6.49, *p* < 0.05. Consistent with the pain ratings, participants reported a reduced urge on choice compared with directed inhibition trials (Choice: *M* = 4.0, *SD* = 1.6; Directed: *M* = 4.5, *SD* = 1.7), *t*_(24)_ = 2.67, *p* < 0.05, while there was no such difference between choice and directed action trials (Choice: *M* = 5.0, *SD* = 1.4; Directed: *M* = 4.6, *SD* = 1.6), *t*_(24)_ = −1.70, *p* = 0.10. The main effect of Belief condition was not significant, *F*_(1, 23)_ = 0.10, *p* = 0.76. Crucially however, there was a significant interaction effect of Belief condition × Instruction, *F*_(1, 23)_ = 6.22, *p* < 0.05. *Post-hoc t*-tests revealed that participants in the anti-free will condition tended to report a stronger urge to press on directed trials than on choice trials, *t*_(11)_ = 2.044, *p* = 0.066, whereas this was not the case for control subjects, *t*_(12)_ = −1.465, *p* = 0.17 (Figure 5). This may be indicative of a greater urge to act when externally instructed on the part of anti-free will participants. Similar results were obtained by Alquist et al. (2013), who found that anti-free will participants conformed more to external pressure.

### Adaptive strategies on choice trials

Based on the hypothesis that anti-free will participants might lack adaptive strategies, we conducted an exploratory analysis in which we investigated whether preceding trial pain or trial type had an influence on response selection during choice trials. We assumed that high pain trials might create a strong incentive to “quit” when subsequently given a choice, thereby activating a strategy that is protective of the organism. Similarly, participants might attempt to create subjectively easier response sequences when granted the opportunity. These strategies would presumably only be present for control participants, as anti-free will participants tend to be less inclined to adjust their behavior to the present situation (Rigoni et al., 2013).

#### Pain on preceding trial

To investigate the influence of pain on subsequent choice behavior, we computed each participant's mean pain rating for the trials preceding choice inhibition and choice action trials. A mixed design ANOVA with factors of Belief condition (Anti-free will vs. Control) and Response (Choice Action vs. Choice Inhibition) was then conducted on mean pain rating for n-1 trials. The analysis revealed no main effects or interactions, *F*s < 0.838, *p*s > 0.36, indicating that pain ratings on the preceding trial did not differ between choice inhibition and choice action trials, for either experimental group. This would suggest that participants do not use recent pain as a factor in deciding whether to act or inhibit when given the choice.

#### Response styles

**Figure 6:**
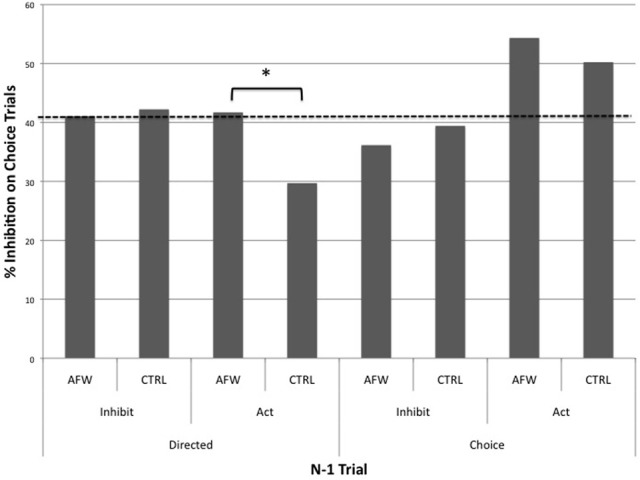
**N-1 trial contribution to response tendencies in each experimental group**. Compared with anti-free will participants (AFW), control participants (CTRL) tend to inhibit less often following a directed press trial. The dashed line indicates the grand mean proportion of inhibition. ^*^p < 0.05.

To investigate response styles, we computed mean proportions of inhibition during choice trials following each of the four trial types. A mixed design ANOVA with factors of Belief condition (Anti-free will vs. Control), n-1 Instruction (Choice vs. Directed), and n-1 Response (Action vs. Inhibition) was then conducted on mean proportion of inhibition in choice trials. This gave an index of how often participants chose to inhibit rather than act following a particular trial type (Figure 6). The analysis revealed a main effect of n-1 Instruction (Choice: *M* = 45.0% inhibition on subsequent choice trial; Directed: *M* = 38.7% inhibition on subsequent choice trial), *F*_(1, 23)_ = 6.366, *p* < 0.05, such that participants tended to choose to inhibit more often following a choice trial. There was also a significant interaction between n-1 Instruction and n-1 Response, *F*_(1, 23)_ = 11.460, *p* < 0.01, such that participants chose to inhibit more often following a choice action trial (*M* = 52.2%) than any other trial type (Choice Inhibit n-1 = 37.9%; Directed Action n-1 = 35.5%; Directed Inhibition n-1 = 41.7%), *t*s > 2.64, *p*s < 0.05. Furthermore, there was a non-significant trend toward an interaction between n-1 Response and Belief condition, *F*_(1, 23)_ = 3.523, *p* = 0.07. Anti-free will participants tended to inhibit more often following an action trial (*M* = 48.0%) than an inhibition trial (*M* = 38.6%), *t*_(11)_ = −2.164, *p* = 0.05, *d* = 0.63, whereas this was not the case for controls (Action n-1: *M* = 40.0%; Inhibition n-1: *M* = 40.8%), *t*_(12)_ = 0.251, *p* = 0.806, *d* = 0.03. This may indicate a more explicit tendency to alternate in an attempt to satisfy the 50% choice instruction. Finally, *post-hoc t*-tests confirmed that the primary difference in proportion of inhibition between experimental groups lay in directed action n-1 trials. Control subjects chose to inhibit significantly less often than anti-free will participants following a directed action trial (Control: *M* = 29.7%; Anti-free will: *M* = 41.7%), *t*_(23)_ = −2.490, *p* < 0.05, *d* = 0.99. This may be indicative of an additional adaptive strategy on the part of control participants, as response repetitions are subjectively less effortful than response switches.

## Discussion

In the present study, we employed a novel experimental approach using thermal pain stimulation in order to demonstrate the moderating nature of high-level beliefs on self-regulation. In particular, we sought to probe whether reducing participants' belief in free will could lead to a form of intentional disengagement that influences selection and inhibition of action within a “hot” motivational system (Metcalfe and Mischel, 1999).

In line with our predictions, participants who were induced to disbelieve in free will were significantly slower to initiate a response on trials in which they chose to act in order to terminate the pain stimulation. This directly corresponds to the hypothesis that anti-free will participants would exhibit less intentional engagement. Interestingly, this effect is only evident when a pain avoidance response has to be executed internally rather than externally, suggesting not a global passivity, but rather a specific impairment in intentional self-regulation. This dissociation is in accordance with previous evidence that intentional and stimulus-driven actions rely on distinct functional (Herwig et al., 2007) and neural (Müller et al., 2007) mechanisms. The amount of slowing on choice action trials was furthermore correlated with the degree of the effectiveness of the belief manipulation, suggesting a direct link between the weakening of free will beliefs and the voluntary management of a behavioral response to an aversive stimulus. This mirrors the finding by Rigoni et al. (2011) in which decreases in the readiness potential were correlated with a change in anti-free will scores.

Moreover, anti-free will participants reported greater urges to terminate the trial when their behavior was guided by the cue compared to when they were able to freely choose, suggesting a disengagement from the task when externally instructed. Importantly, and in contrast with previous studies (e.g., Kühn et al., 2009; Rigoni et al., 2012), the aforementioned differences are not confounded by differential response biases, as the proportion of inhibition in choice trials was equivalent between control and anti-free will participants.

Our analysis of potentially adaptive strategies revealed surprising results. Participants do not appear to use recent pain as a criterion in deciding whether to act or inhibit when given the choice. However, we do find differences between the experimental groups in terms of their response styles. Interpretations are merely speculative at this point, but it seems plausible that this effect could be related to minimizing cognitive effort (e.g., Kool et al., 2010). For instance, one could suppose that control participants select a subjectively easier strategy when exhibiting a bias to repeat an action response (e.g., Mayr and Bell, 2006). On the other hand, one could interpret the anti-free will participants as selecting the less effortful strategy, by avoiding two (subjectively more painful) inhibition trials in a row (e.g., law of least effort, Hull, 1943). In the future, this could be disentangled by presenting blocks composed solely of choice trials in order to determine, via longer choice trial sequences, which is the favored strategy: response repetitions or avoidance of effortful combinations.

Taken together, the present study supports and extends previous research on intentional inhibition (Brass and Haggard, 2007; Kühn et al., 2009; Filevich et al., 2012; Rigoni et al., 2012). In particular, it is the first to investigate voluntary inhibition of behavior in an ecologically valid experimental setting that involves hot motivational systems rather than entirely arbitrary choices. Participants reported less pain and a reduced urge to terminate the trial on choice inhibition trials compared with directed inhibition trials, while choice and directed press trials were more comparable. Thus, the pain paradigm we introduce offers an effective way to dissociate between voluntary and instructed inhibition on a behavioral level, which opens the door to new ways of investigating inhibition in which behaviorally-relevant options are available to the participant.

That being said, as this study served as a first pilot of a novel paradigm, our investigation must be seen as exploratory in nature, and our conclusions considered accordingly. The exclusion of participants who did not experience sufficient pain levels is an unfortunate limitation of the present line of research (Supplementary Material includes a summary of the excluded participants' results, for a comprehensive overview of our findings). Future studies should endeavor to ensure that a sufficient pain tolerance threshold is obtained for each participant, or that unsuitable participants are excluded in advance of testing. This may require rigorous pre-testing of criteria such as whether participants are able to reliably report their tolerance thresholds, and whether or not they adapt too quickly to pain over the course of the experiment.

On a larger scale, the observed effects also exemplify a growing body of research that reveals the influence of higher-order beliefs and metacognitions on behavioral control. As discussed earlier, determinist beliefs have been shown to have an effect on prosocial behavior (Vohs and Schooler, 2008; Baumeister et al., 2009, 2011), basic motor and cognitive processes (Rigoni et al., 2011, 2013), intentional inhibition (Rigoni et al., 2012), and now on self-regulation of a “hot” incentive response system (Morsella, 2005). Yet free will beliefs are not the only higher-order cognitions capable of influencing a variety of processes underlying behavioral control.

For instance, one factor that has been proposed to have a strong influence on self-control is “ego depletion,” or the phenomenon in which exertion of self-control exhausts a common regulatory resource, leading to hindered performance on subsequent tasks (Muraven et al., 1998; Vohs et al., 2008; Baumeister et al., 2009; Hagger et al., 2010). However, recent research has revealed that participants' relevant belief systems are likely to be more crucial than actual depletion when it comes to self-regulatory capacity. For instance, Job et al. (2010) demonstrated that only participants who thought of willpower as a limited resource demonstrated the typical pattern of ego depletion, while the effect was completely absent in participants who lacked this conviction. Similarly, Clarkson et al. (2010) found that regardless of how depleted participants actually were, if they perceived themselves as less depleted, they failed to demonstrate ego depletion effects during subsequent task performance (see also Vohs et al., 2012).

These observations indicate that beliefs regarding regulatory resources are distinct from the resources themselves, and can impact task performance independently. The present study complements this line of research. There is little incentive for engagement in self-control under the assumption that behavior is fully determined, and in this way free will beliefs are able to influence the decision to exert regulatory effort. Accordingly, assumptions about the existence of free will can be considered as operating in parallel with beliefs about regulatory capacities. The former speaks to one's motivation to engage in self-regulation, while the latter informs one's available resources for self-control. Moreover, while the aforementioned ego depletion studies have examined task-relevant beliefs as stable traits, here we demonstrate the relevance of lay beliefs more directly by manipulating them experimentally. Our findings therefore indicate that the impact of higher-order beliefs on self-regulatory engagement is not limited to stable, trait-like effects, but that even subtle state-like fluctuations in the strength of beliefs can affect the amount of effort that people invest in self-control. A fundamental belief in control over one's actions may therefore prove to be an integral prerequisite for self-regulatory investments. Future studies should more directly investigate the mechanisms by which higher-order beliefs impact the recruitment of self-control.

## Conflict of interest statement

The authors declare that the research was conducted in the absence of any commercial or financial relationships that could be construed as a potential conflict of interest.

## Supplementary material

The Supplementary Material for this article can be found online at: http://www.frontiersin.org/Cognition/10.3389/fpsyg.2013.00614/abstract
