# Supplementary material for: The influence of high-level beliefs on self-regulatory engagement: evidence from thermal pain stimulation
Source: Front Psychol. 2013 Sep 23;4:614. doi: 10.3389/fpsyg.2013.00614 (PMC3779819; doi:10.3389/fpsyg.2013.00614)
Supplement: Supplementary file 1 [file DataSheet1.DOCX]

**APPENDIX 1**

**Anti-Free Will Statements**

Cluster 1: Anti-Free Will

1. Science has proven that people have no free will.
2. I am not always free to do as I please.
3. People often state that they have free will, but in fact the only thing they experience is that their brain has made a decision.
4. Scientists have shown that free will is a trick of the mind.
5. People have no free will.
6. People are not capable of choosing their actions freely.
7. You usually aren’t capable of controlling the decisions you make.
8. Science has proven that free will, as we know it, doesn’t exist.
9. I think that I make most choices consciously, but actually that is nothing but an illusion.
10. A person might think they have free will, but in fact their brain makes each decision before they became conscious of making it.
11. People sometimes think they have chosen something consciously, when in fact they had no choice to begin with.
12. People don’t make decisions out of free will, but as the result of an interplay of factors.
13. People think they are free in making their choices, when in fact their choices are determined by a number of different physical and environmental factors.
14. No matter how much willpower you have, you can never conquer your body’s needs.
15. Social conditions limit my ability to act freely.
16. Even when you make bad choices, you usually aren’t fully responsible for them.
17. People who have made a mistake often couldn’t have done differently.
18. Mental disorders that arise in healthy people show us that people don’t always have a choice to do what they want.
19. A person can’t be held fully responsible for all the choices that he or she makes.
20. No one can fully control their actions, and therefore you can’t be fully held responsible for your behavior.

Cluster 2: Scientific Determinism

1. People’s decisions are mostly biologically determined.
2. A person’s early environment determines their intelligence and personality.
3. All human behavior will eventually be explained based on experiences and biological determination.
4. Science has shown that what talents you have are usually biologically determined.
5. The environment in which you grew up in as a child will determine your success as an adult.
6. Someone is intelligent when he is biologically predestined to be so.
7. Every behavior is determined by brain activity, which is in turn determined by a combination of environmental and genetic factors.
8. How wealthy I am, as well as what race and gender I am, will determine the decisions I am able to make.
9. Alcoholism and other behavioral disorders are caused by genetic factors.
10. People that exhibit bad conduct do so because of their earlier experiences.
11. Human behavior follows the laws of nature.
12. Biological and environmental influences determine the decisions people make.
13. A large part of human behavior is biologically determined and can be considered as instinctive.
14. A person’s genes will determine their future.
15. Genetic differences between Asians and Europeans are the cause of most differences in skills and behavior between both groups.
16. The personality of parents will largely determine the personality of their children.
17. People are genetically predestined to become who they end up being.
18. Our mental activity is determined solely by physical processes.
19. A person’s biological constitution will determine their talents and personality.
20. Scientists have shown that your previous experiences strongly determine your current choices.

Cluster 3: Fatalistic Determinism

1. You can’t change your destiny, no matter how hard you try.
2. What will be will be, and you can’t change that.
3. There are higher powers at work that determine a person’s destiny.
4. What needs to happen, will happen.
5. A person’s fate is determined and will constrain their future.
6. Mysterious forces determine what happens to us.
7. Fate determines what could possibly happen in my life.
8. Mysterious forces seem to influence how my life ends up.
9. The moment at which you will die is pre-determined.
10. Higher powers determine how life turns out.
11. If everything goes wrong for me, that is because I wasn’t born lucky.
12. Some people are less lucky than others because they are pre-destined to be so.
13. You cannot change what must happen.
14. Your future is pre-determined.
15. Fate plays an important role in a person’s future.
16. Your future is largely determined by chance.
17. Higher powers determine what we end up doing with our lives.
18. I can’t change anything about what life will have to offer for me.
19. My life path is pre-determined.
20. Faith determines my past, present and future.

**Neutral Statements**

1. The Nile River in Africa is the longest river in the world.
2. King butterflies are slow fliers, but they are seen at sea up to hundreds of miles from land.
3. Over 30 million people in America have a diastema (a gap between their front teeth).
4. A spring chicken is a very young chicken or rooster that weighs no more than 500 grams.
5. The Olympic games are held every four years.
6. Buyers of secondhand cars and new cars may think each other crazy, but in fact they need one another.
7. All sturgeon prawns are male in their first year of life.
8. An ant will only breathe once every two minutes.
9. The most poisonous animal on earth is a yellow-black frog that weighs 1 gram.
10. Ammonite fossils are spiral and used to house a squid-like creature.
11. Your hearing decreases slightly when you eat too much.
12. Leonardo De Vinci invented the hand scissors.
13. On a summer’s day the land heats up faster than the water.
14. Every year a large number of people are hit by an influenza virus that is active for 3 to 5 days.
15. The word marathon stems from the Greek city of Marathon, 40 kilometers away from Athens.
16. Goldfish will lose their color when they stay in a dark or mildly lit room.
17. The average life span of an umbrella is one and a half years.
18. The Mexican salamander species Axolotl can regenerate a cut-off tail and even parts of their heart.
19. Despite the long neck of the giraffe, they have just as many neck vertebrae as humans.
20. The oldest tree in the world is approximately 4767 years old and is a bristlecone pine in the White Mountains of California.
21. Sugar beets and sugar cane are grown in over 100 countries.
22. Pocket calculators only came into use after 1970.
23. Success should not be measured solely by accomplishments, but also by the obstacles that were conquered.
24. Nearly half the bones in your body are in your hands and feet.
25. You will burn 150 calories per hour by banging your head against the wall.
26. There are certain species of bamboo that can grow more than one meter overnight.
27. People who eat a banana at night have a better chance to fall asleep.
28. Most appliances are under warranty for a full year for all kinds of defects.
29. The construction of a building needs to occur very slowly and with lots of support.
30. The smallest people in the world live not in Asia, but in Zaïre (Africa).
31. It is better to be governed by a king than by a president.
32. A female oyster can give birth to up to 100 million young.
33. In the sport of clay pipe smoking the goal is to keep a clay pipe burning as long as possible.
34. Men get the hiccups more often than women.
35. The oldest and most well-known Christmas market is held every year in Nürnberg, Germany.
36. An ostrich’s eye is larger than its brain.
37. Running inside is easier than running outside.
38. Most mountain climbers know that for every 150 meters that you climb it will get approximately one degree colder.
39. People often judge one another’s inner world on the basis of their appearance.
40. Sunbeams can only penetrate the earth for a couple of centimeters and will only heat up the top layer.
41. Inventories are made in all the large companies to calculate what they have in stock and what needs to be bought.
42. Water is the only substance that can be found on earth in its natural environment in three forms: solid, liquid, or gas.
43. The width of a Boeing 747 exceeds that of the first flight of the Wright brothers.
44. If you eat dandelions, you will need to pee more.
45. Alkaline power cells usually live longer than ordinary batteries.
46. We each have about 400 different beneficial bacteria in our intestines.
47. Every hour about 1 billion cells in your body need to be replaced.
48. The platypus and the spiny anteater are the only animals in the world that lay eggs and suckle their young.
49. Human hair grows about 15 centimeters a year on average.
50. A clay pigeon is a disc of clay that is shot into the air from a place you can’t see.
51. The average American eats about half a ton of cheese during their lifetime.
52. Most things of value can’t be bought with money.
53. A person’s heart pumps blood through their body with an exceptional power.
54. A party with a lot of people is more pleasant that a party with only few people.
55. Hearing aids are becoming more and more fashionable.
56. Being reachable everywhere means the same as being able to be disturbed at any time.
57. Being beautiful is subjective.
58. When you mix plaster with water you get a kind of paste with which you can make imprints.
59. It is better to close down shops on Saturdays and to open up on Sundays.
60. The earth has a circumference of about 40000 kilometers.
