# Supplementary material for: The influence of high-level beliefs on self-regulatory engagement: evidence from thermal pain stimulation
Source: Front Psychol. 2013 Sep 23;4:614. doi: 10.3389/fpsyg.2013.00614 (PMC3779819; doi:10.3389/fpsyg.2013.00614)
Supplement: Supplementary file 2 [file DataSheet2.DOCX]

**APPENDIX 2**

**Probe Questions**

Indicate whether you had to remember this statement: 1 = yes 2 = no

ANTI-FREE WILL STATEMENTS:

1. A person’s biological constitution determines their talents and personality.
2. People make their decisions primarily on the basis of their biological intelligence.
3. People often state that they have free will, but in fact they only experience their brain having made a decision.
4. One's fate is fixed in advance and determines their future.
5. Science has demonstrated that people have no free will.
6. The concept of fate negates what will happen during the course of one’s life.

NEUTRAL STATEMENTS:

1. A spring chicken is a very young chicken or rooster that weighs more than 500 grams.
2. The platypus and the spiny anteater are the only mammals in the world that lay eggs and suckle their young.
3. Ammonite fossils are spiraled and used to house a squid-like creature.
4. The smallest people in the world live not in Asia, but in Africa (Tanzania).
5. The most poisonous animal on earth is a green-black frog that weighs 1 gram.
6. Most mountain climbers know that for every 150 meters one climbs, it becomes approximately one degree colder.
