# Supplementary material for: The influence of high-level beliefs on self-regulatory engagement: evidence from thermal pain stimulation
Source: Front Psychol. 2013 Sep 23;4:614. doi: 10.3389/fpsyg.2013.00614 (PMC3779819; doi:10.3389/fpsyg.2013.00614)
Supplement: Supplementary file 3 [file DataSheet3.DOC]

**APPENDIX 3**

**Results Including Low Pain Participants**

***Overview***

The ‘low pain’ group consisted of 23 participants (4 males), with 12 participants in the anti-free will condition and 11 in the control condition. Total FAD-scores did not differ significantly between the low and high pain group, t(46) = 0.77, p=.44. The grand mean pain rating for the low pain group was 3.7 (*SD* = 0.57). The below results include all participants, and treat Pain Group as a between-subjects factor.
***Behavioral analyses***

***Reaction times.*** On trials in which participants were cued to button press, participants performed the correct response in nearly all trials (*M* = 98%, *SD* = 3%). We expected anti-free will participants to be significantly slower than controls, particularly on choice trials. A mixed design ANOVA on RTs with Instruction (Choice vs. Directed) as a within-subjects factor, and Belief condition (Anti-free will vs. Control) and Pain Group (High vs. Low) as between-subjects factors, revealed a main effect of Instruction, *F*(1,44) = 139.31, *p* < .001, such that participants were slower to respond on choice trials (Choice: *M* = 795 ms, *SD* = 21 ms; Directed: *M* = 563 ms, *SD* = 15 ms), consistent with piloting and reflecting the time needed for a response decision. Neither the main effect of Belief condition nor the main effect of Pain Group were significant, *p*s *>* .31**.** Crucially however, there was a significant three-way interaction effect of Instruction x Belief Condition x Pain Group, *F*(1,44) = 4.43, *p* < .05. Planned comparisons revealed a significant RT difference between anti-free will participants and controls on choice action trials in the high pain group, *t*(23) = - 2.07, *p* < .05, such that anti-free will participants were significantly slower to respond when given a choice (*M* = 871 ms, *SD* = 133 ms) than controls (*M* = 748 ms, *SD* = 162 ms). No such effect was found on exogenous action trials, *t*(23) = -.69, *p* = .497 (Anti-free will: *M* = 582 ms, *SD* = 94 ms; Control: *M* = 552 ms, *SD* = 121 ms). In the low pain group there were no significant RT differences between the Belief Conditions, *p*s>.40.

***Correlation of FAD difference scores with choice reaction times.*** To examine the relationship between participants’ RTs and free will beliefs more thoroughly, we performed an additional correlation analysis. The aim of this analysis was to test to what extent the slowed responding on endogenous action trials was related to the effectiveness of the belief manipulation. To this end, we first computed each participant’s change in anti-free will beliefs, across experimental condition, by subtracting participants’ post-experimental scores on the anti-free will subscale of the FAD from their pre-experimental scores. Second, we computed a difference score of participants’ mean RTs on endogenous and exogenous action trials to create an index of each participant’s decision time at pushing the button. There was a significant positive correlation between the two difference scores for participants in the high pain group, *r*(25)=0.40, *p* < .05, reflecting that those subjects who showed a stronger reduction in free will beliefs were also slower to make the decision to press the button. In line with the previous analysis, no such effect was observed for participants in the low pain group, *r*(23)=0.04, *p* = .87.

***Proportion of inhibition on choice trials.*** On trials in which participants were cued to choose between pressing and inhibiting, participants opted to inhibit in 44.92% of all trials (*SD* = 12.60%).

The proportion of inhibition on choice trials was analyzed in an ANOVA with Belief condition and Pain Group as between-subjects factors. This analysis revealed a main effect of Pain Group, *F*(1,44) = 3.87, *p* = .05, such that participants in the high pain group chose to inhibit less often than participants in the low pain group (high pain: *M* = 41.6%, *SD* = 2.5%; low pain: *M* = 48.6%, *SD* = 2.6%). However, there was no interaction effect, nor a significant main effect of Belief Condition, ps>. 70. A planned comparison revealed no significant difference between anti-free will participants (*M* = 42.43%, *SD* = 10.22%) and controls (*M* = 40.59%, *SD* = 9.64%) in the high pain group, *t*(23) = -.462, *p* = .65. This lack of a difference between experimental groups, which is in contrast to the findings of Rigoni et al. (2012), may be due to the experimental design, which, unlike previous studies, discourages response biases by using an equal proportion of directed press and inhibit trials.

***Ratings***

***Pain ratings.*** Pain ratings were computed for the first and second halves of the experiment to ensure that participants did not adapt to the pain stimulation over the course of the experiment. No differences in pain ratings were observed between the trials of the first and the second half of the experiment (First half: *M* = 4.6, *SD* = 1.1; Second half: *M* = 4.6, *SD* = 1.2), *t*(47) = 0.21, *p* = .84. Pain ratings were analyzed in a mixed design ANOVA using Belief condition as a between-subjects factor, and Response (Action vs. Inhibition) and Instruction as within-subject factors. The main effect of Belief condition was not significant (Anti-free will: *M* = 4.5, *SD* = 1.1; Control: *M* = 4.7, *SD* = 1.2), *F*(1,46) = 0.23, *p* = .63, reflecting that subjective pain across trials was equivalent for the two groups. However, there was a significant main effect of Response (Action: *M* = 4.2, *SD* = 0.2; Inhibition: *M* = 5.0, *SD* = 0.1), *F*(1,46) = 51.81, *p* < .001, indicating higher perceived pain on inhibition compared with action trials, presumably due to the lengthier pain stimulation. Moreover, there was a main effect of instruction, *F*(1,46) = 4.77, *p* < .05. Participants had higher pain ratings on directed than on choice trials (Directed: *M* = 4.7, *SD* = 0.2; Choice: *M* = 4.6, *SD* = 0.2). We also observed an interaction effect of Response x Instruction, *F*(1,46) = 20.12, *p* < .001, reflecting that inhibition trials were rated as less painful when they were chosen rather than directed (Choice: *M* = 4.8, *SD* = 1.1; Directed: *M* = 5.2, *SD* = 0.9), *t*(47) = 5.78, *p* < .001, while action trials were rated as less painful when they were directed rather than chosen (Directed: *M* = 4.1, *SD* = 1.4; Choice: *M* = 4.3, *SD* = 1.4), *t*(47) = -2.30, *p* < .05. Importantly, the lack of a difference between the mean pain ratings of anti-free will and control participants suggests that our findings are not solely due to differences in the overall subjective experience of pain.

***Urge ratings.*** Participants reported a grand mean urge rating of 3.6 (*SD* = 1.7). Participants’ mean urge ratings were higher in the high pain group (*M* = 4.5, *SD* = 1.38) than in the low pain group (*M* = 2.6, *SD* = 1.37), *t*(46) = 4.94, p < .001. Urge ratings were analyzed with a mixed design ANOVA akin to that of pain ratings. The analysis revealed a significant main effect of response, reflecting greater urges on action trials than inhibition trials (Action: *M* = 4.0, *SD* = 0.3; Inhibition: *M* = 3.2, *SD* = 0.3), *F*(1,46) = 8.46, *p* < .05. There was also a significant interaction effect of Response x Instruction*, F*(1,46) = 9.74, *p* < .05. Consistent with the pain ratings, participants reported reduced urges on choice compared with directed inhibition trials (Choice: *M* = 3.0, *SD* = 1.7; Directed: *M* = 3.4, *SD* = 1.9), *t*(47) = 2.89, *p* < .05, while this effect was reversed for choice and directed action trials (Choice: *M* = 4.2, *SD* = 2.1; Directed: *M* = 3.7, *SD* = 2.2), *t*(47) = -2.27, *p* < .05. The main effect of Belief condition was not significant (Anti-free will: *M* = 3.7, *SD* = 1.6; Control: *M* = 3.5, *SD* = 1.8), *F*(1,46) *=* 0.14 , *p* = .72. Notably however, we observed a trend towards a three-way interaction effect of Belief condition x Response x Instruction, *F*(1,23) = 3.36, *p* = .07. Post-hoc t-tests revealed that participants in the control condition tended to report a stronger urge to press on choice action trials than on directed action trials (Choice Action: *M* = 4.3, *SD* = 2.1; Directed Action: *M* = 3.4, *SD* = 2.0), *t*(23) = 4.32, *p* < .001, whereas this was not the case for anti-free will subjects (Choice Action: *M* = 4.2, *SD* = 2.1; Directed Action: *M* = 4.1, *SD* = 2.4), *t*(23)= 0.21, *p*=.84. Though strong conclusions cannot be drawn from this non-significant result, it may be indicative of a greater urge to act when externally instructed on the part of anti-free will participants. Similar results were obtained by Alquist et al. (2013), who found that anti-free will participants conformed more to external pressure.
